# Diagnostic gastrointestinal markers in primary lung cancer and pulmonary metastases

**DOI:** 10.1007/s00428-023-03583-w

**Published:** 2023-06-22

**Authors:** Karina Malmros, Andreas Lindholm, Halla Vidarsdottir, Karin Jirström, Björn Nodin, Johan Botling, Johanna S. M. Mattsson, Patrick Micke, Maria Planck, Mats Jönsson, Johan Staaf, Hans Brunnström

**Affiliations:** 1 Division of Pathology, Department of Clinical Sciences Lund, Lund University, SE-221 00 Lund, Sweden; 2 Department of Genetics and Pathology, Laboratory Medicine Region Skåne, SE-205 02 Malmö, Sweden; 3 Department of Surgery, Landspitali University Hospital, Hringbraut, 101, Reykjavik, Iceland; 4 Division of Oncology and Therapeutic Pathology, Department of Clinical Sciences Lund, Lund University, SE-221 00 Lund, Sweden; 5 Department of Genetics and Pathology, Laboratory Medicine Region Skåne, SE-221 85 Lund, Sweden; 6 Department of Immunology, Genetics and Pathology, Uppsala University and Uppsala University Hospital, SE-751 85 Uppsala, Sweden; 7 Division of Oncology, Department of Clinical Sciences Lund, Lund University, Medicon Village, SE-223 81 Lund, Sweden; 8 Division of Respiratory Medicine, Allergology, and Palliative Medicine, Department of Clinical Sciences Lund, Lund University, SE-221 85 Lund, Sweden; 9 Division of Translational Cancer Research, Department of Laboratory Medicine, Lund University, Medicon Village, SE-223 81 Lund, Sweden

**Keywords:** A33, Cadherin 17, CDX2, CK20, Immunohistochemistry, MUC2, SATB2

## Abstract

Histopathological diagnosis of pulmonary tumors is essential for treatment decisions. The distinction between primary lung adenocarcinoma and pulmonary metastasis from the gastrointestinal (GI) tract may be difficult. Therefore, we compared the diagnostic value of several immunohistochemical markers in pulmonary tumors. Tissue microarrays from 629 resected primary lung cancers and 422 resected pulmonary epithelial metastases from various sites (whereof 275 colorectal cancer) were investigated for the immunohistochemical expression of CDH17, GPA33, MUC2, MUC6, SATB2, and SMAD4, for comparison with CDX2, CK20, CK7, and TTF-1. The most sensitive markers for GI origin were GPA33 (positive in 98%, 60%, and 100% of pulmonary metastases from colorectal cancer, pancreatic cancer, and other GI adenocarcinomas, respectively), CDX2 (99/40/100%), and CDH17 (99/0/100%). In comparison, SATB2 and CK20 showed higher specificity, with expression in 5% and 10% of mucinous primary lung adenocarcinomas and both in 0% of TTF-1-negative non-mucinous primary lung adenocarcinomas (25–50% and 5–16%, respectively, for GPA33/CDX2/CDH17). MUC2 was negative in all primary lung cancers, but positive only in less than half of pulmonary metastases from mucinous adenocarcinomas from other organs. Combining six GI markers did not perfectly separate primary lung cancers from pulmonary metastases including subgroups such as mucinous adenocarcinomas or CK7-positive GI tract metastases. This comprehensive comparison suggests that CDH17, GPA33, and SATB2 may be used as equivalent alternatives to CDX2 and CK20. However, no single or combination of markers can categorically distinguish primary lung cancers from metastatic GI tract cancer.

**Supplementary Information:**

The online version contains supplementary material available at 10.1007/s00428-023-03583-w.

## Introduction

Histopathological diagnosis of pulmonary tumors is essential for informed treatment decisions. Treatment predictive testing and subsequent choice of therapeutic regimen differ between histological lung cancer subtypes. Even more important is the accurate diagnosis of metastatic cancer, with the lungs being a common site of metastases, as treatment and predictive testing are fundamentally different compared to primary lung cancer.

The distinction between primary lung adenocarcinoma (AC) and pulmonary AC metastases originating from the gastrointestinal (GI) tract may be difficult based on morphology alone [1, 2]. Immunohistochemical (IHC) staining, with a panel of markers, is often of great value to determine tumor origin. However, the traditional lung AC marker thyroid transcription factor 1 (TTF-1) is negative in 11–30% of all lung AC [3–6] and, notably, in the majority of mucinous lung AC [7–9]. In addition, the commonly used GI markers cytokeratin (CK) 20 and caudal-type homeobox 2 (CDX2) are often positive in these mucinous subtypes, thus not being sufficiently specific for GI origin. Several studies have analyzed these IHC markers in pulmonary tumors and mucinous AC from various sites [10–14]. Due to the overlapping expression, it is accepted that “there is no useful marker to differentiate pulmonary mucinous adenocarcinoma from metastatic mimics” [2].

Therefore, alternative GI markers, such as cadherin 17 (CDH17) and glycoprotein A33 (GPA33), have been suggested as possible solutions to close this crucial diagnostic gap. For CDH17 [15–18] and GPA33 [19, 20] moderate (upper GI tract AC) to high (colorectal AC) sensitivity in combination with high specificity have been reported. Also, special AT-rich sequence-binding protein 2 (SATB2) has demonstrated high sensitivity and specificity for colorectal AC [17, 21–24]. The MUC family has been regarded as promising candidates as well. While MUC2 has been suggested to be specific for the GI tract and mainly expressed in colorectal cancer [8, 25, 26], especially mucinous colorectal AC [27], MUC6 has been reported to be positive more often in upper GI tract and pancreatic AC [28–31]. Finally, loss of nuclear [32] or loss of both nuclear and cytoplasmic [33] expression of mothers against decapentaplegic homolog 4 (SMAD4), but also positive nuclear SMAD4 staining [34], has been reported to be of potential value to distinguish mainly pancreatic cancer from non-GI cancers.

Although there are several studies in the literature, the markers have often been analyzed as single markers or compared with a few selected markers. Furthermore, their performance has not been tested in a broad range of pulmonary metastases. Thus, comparisons of these new markers in a comprehensive tissue material are missing.

The present study aimed to investigate the diagnostic value of the novel GI tract markers compared with traditional markers in pulmonary tumors using large non-selective cohorts of resected carcinomas including both primary lung cancers and metastases to the lungs.

## Material and methods

### Study population

The present study was conducted using tissue microarrays (TMA) from four cohorts with resected primary lung cancers or resected pulmonary metastases previously described in detail and investigated for other diagnostic IHC markers [4, 13, 35, 36]. Compared to the previous studies on the same cohorts, sufficient tumor tissue for assessment was missing in the TMA blocks for a few cases, while for one cohort (the Southern Swedish Lung Cancer Study), 11 cases not evaluated in former studies were included in the present investigation. Previous successive addition of diagnostic markers [4, 13, 35, 36] has resulted in revised subtyping for a few primary lung cancers compared to early publications on the material [37]. In the present study, diagnoses were in accordance with the current WHO classification [1]. Staining of whole tissue slides for selected markers and cases has previously been performed to rule out, e.g., focal TTF-1 positivity (for diagnostics of large-cell carcinoma) [37]. In the clinical setting, the cases had typically been discussed at multidisciplinary team meetings. In the present study, “mucinous lung AC” included the subtypes invasive mucinous AC (both pure and mixed non-mucinous/mucinous), mucinous minimally invasive AC, enteric-type AC, and colloid AC.

The cohorts, with the number of evaluable cases in the present investigation, are here described in brief. The Uppsala Non-Small-Cell Lung Cancer Study is a retrospective study with 326 evaluable consecutive resected lung cancers from 322 individuals surgically treated in 2006–2010. The Southern Swedish Lung Cancer Study is a prospective non-selective study including 202 resected lung cancers from 199 individuals from 2005 to 2011. The Malmö Diet and Cancer Study is a population-based study on incident cancer including 112 resected lung cancers from 110 individuals from 1992 to 2010. Eleven patients were included in both the Southern Swedish Lung Cancer Study and the Malmö Diet and Cancer Study, and each of these was only included once in the present study. Hence, 629 resected primary lung cancers from 620 individuals (9 cases with two synchronous primary lung cancers each) were included in the present investigation. In all three lung cancer cohorts, cases with neoadjuvant treatment were excluded, and in the Uppsala cohort and the Southern Swedish Lung Cancer Study carcinoid tumors were excluded as well, while also small-cell lung cancers were excluded in the Uppsala cohort.

The metastasis cohort included 422 consecutive resected epithelial lung metastases of non-pulmonary origin from 341 individuals surgically treated at the Skåne University Hospital, Lund, between 2000 and 2014. Tissue cores were taken from each pulmonary metastasis in cases where multiple metastases had been resected (range 1–3 metastases per individual in the study population).

For the four included cohorts, two 1-mm tissue cores were taken from each tumor except for the Southern Swedish Lung Cancer Study cohort where three cores were taken from each tumor.

### Immunohistochemical staining

Details for the applied IHC markers are found in Table 1. Existing data for CK7, CK20, CDX2, and TTF-1 from our previous publications were used in the present study for comparison with the investigated markers [4, 13]. As in former studies, 4-μm-thick tissue sections from the TMAs were automatically pretreated and stained on a Ventana BenchMark Ultra using the Ventana ultraView Universal DAB Detection Kit (Ventana Medical Systems, Tucson, AZ) at the Department of Pathology Lund/Malmö (identical to the clinical diagnostic procedures at the Skåne University Hospital). Control tissue was used on all slides (see Table 1 and images in Supplementary Figure 1). Also, all slides contained TMA cores with lung parenchyma and bronchiole (functioning as an internal positive control for the previously annotated markers CK7, napsin A, and TTF-1). All slides were evaluated by the same pathologist working daily with thoracic pathology (H. B.). Except for SMAD4, the fraction of positive viable tumor cells was scored using the scale: 0, less than 1%; 1, 1–9%; 2, 10–24%; 3, 25–49%; and 4, 50% or more. A score of 2 or more (i.e., at least 10% positive tumor cells) was considered a positive staining result except that a score of 1 was also considered positive for TTF-1 in line with international guidelines [1]. For SMAD4, the cases were instead scored as 0, negative/loss of nuclear and cytoplasmic expression; 1, preserved cytoplasmic positivity only (loss of nuclear expression); or 2, preserved nuclear positivity with/without cytoplasmic positivity, as focal staining did not exist. For all markers, weak to strong staining was considered positive. Nuclear expression of GPA33, as seen in some primary lung cancers and pulmonary metastases of various types, was disregarded. Special care was taken not to interpret the staining of benign cells (such as membranous GPA33 in some alveolar macrophages and cytoplasmic SMAD4 in salivary glands) as positive tumor cells. If data were missing for a marker, a case was excluded only for that marker when calculating the frequency of positivity.

**Table 1:** Details for the immunostains

Antibody	Clone	Vendor	Dilution	Pre-treatment	Control tissue (multi-block)	Evaluated pattern
CDH17	1H3	Sigma-Aldrich	1:5000	CC1	Cervix, colon, small intestine	Membranous
CDX2^a^	EPR2764Y	Ventana	RTU	CC1 + Amp	Pancreas and small intestine, or appendix, liver, tonsil	Nuclear
CK7^a^	SP52	Ventana	RTU	CC1	Appendix, liver, tonsil	Cytoplasmic
CK20^a^	SP33	Ventana	RTU	CC1	Appendix, liver, tonsil	Cytoplasmic
GPA33	EPR4240	Abcam	1:800	CC2	Cervix, colon, small intestine	Membranous
MUC2	CCP58	Dako	1:100	CC1	Appendix, pancreas	Cytoplasmic
MUC6	MRQ-20	Ventana	RTU	CC1 + Amp	Appendix, pancreas	Cytoplasmic
SATB2	EP281	Cell Marque	1:100	CC1	Appendix, liver, tonsil	Nuclear
SMAD4	B8	Santa Cruz	1:20	CC1 + Amp	Cervix, colon, small intestine	Nuclear and cytoplasmic
TTF-1^a^	8G7G3/1	Ventana	RTU	CC1 + Amp	Thyroid plus either placenta or kidney and tonsil	Nuclear

*Amp*, amplification; *CC1*, cell conditioning 1 (EDTA, pH 8); *CC2*, cell conditioning 2 (citrate, pH6); *CDH17*, cadherin 17; *CDX2*, caudal-type homeobox 2; *CK*, cytokeratin; *GPA33*, glycoprotein A33; *MUC*, mucin; *RTU*, ready-to-use; *SATB2*, special AT-rich sequence-binding protein 2; *SMAD4*, mothers against decapentaplegic homolog 4; *TTF-1*, thyroid transcription factor-1

^a^CDX2, CK7, CK20, and TTF-1 stained as part of previous studies [4, 13, 37]

For the primary lung cancers, the two different histological components were evaluated and presented separately for adenosquamous carcinomas and combined large-cell neuroendocrine carcinomas (LCNEC) whenever both components were evaluable in the TMAs. In these cases, the AC component was grouped with “pure” AC cases, etc. Regarding the pulmonary metastases, only the AC component was present in the pulmonary metastases of cervical adenosquamous carcinoma. For tumors with intermingled cell populations (e.g., adenoid cystic carcinoma and malignant myoepithelioma) all tumor cells were evaluated as a single component, except thymomas where only the epithelial cells were evaluated.

## Results

**Fig. 1:**
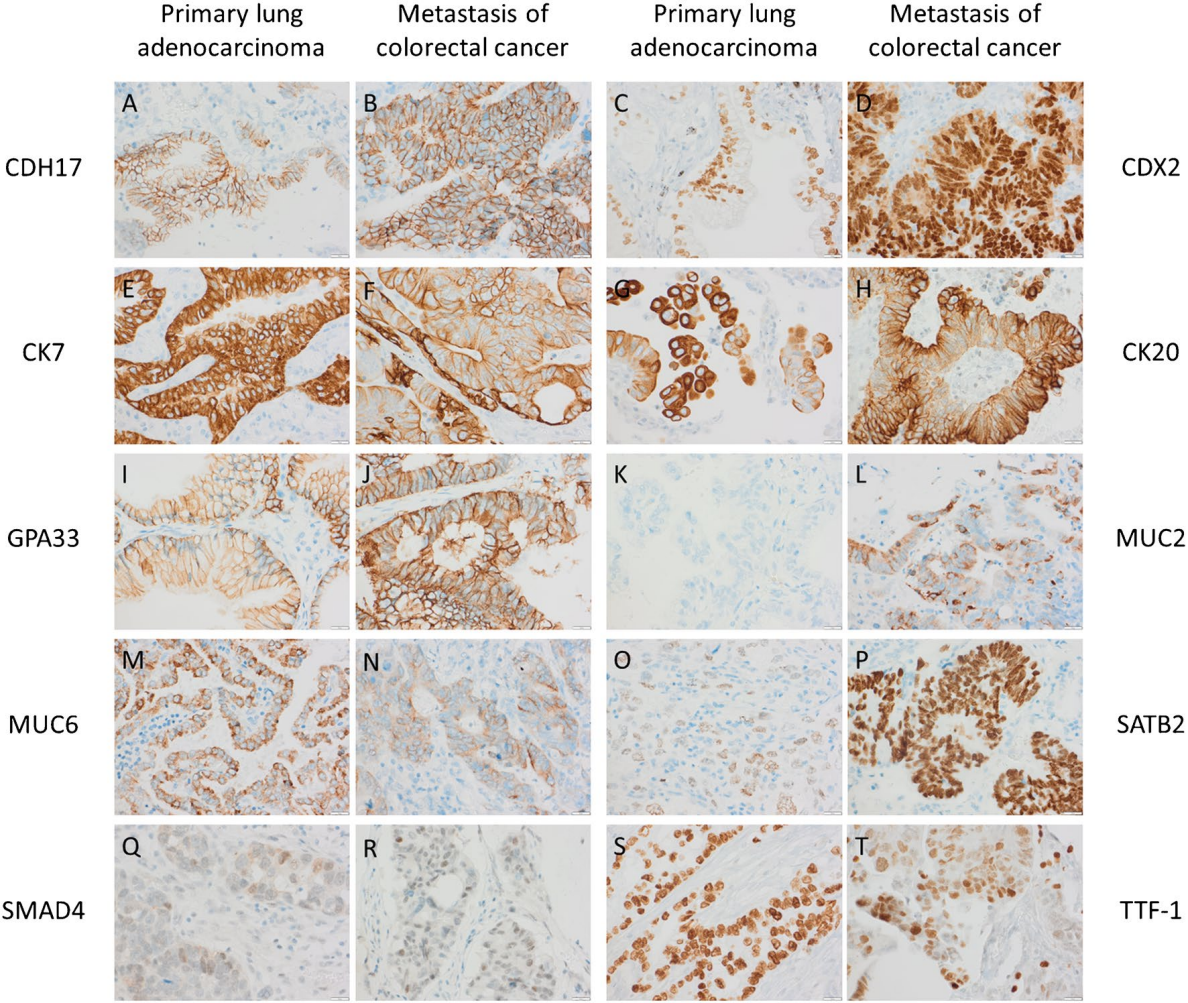
Representative immunohistochemical staining of positive primary lung adenocarcinomas (**A, C, E, G, I, K, M, O, Q, S**) and pulmonary metastases of colorectal cancers (**B, D, F, H, J, L, N, P, R, T**). Note that no MUC2-positive lung cancer case existed (**K**). Also note the strong intensity of CK7 and TTF-1 in pneumocyte bordering or close to the metastatic tumor cells (**F**, **T**), and that large-cell neuroendocrine carcinomas exhibited stronger SATB2 positivity than lung adenocarcinomas (not shown). Scale bar is 20 μm

**Fig. 2:**
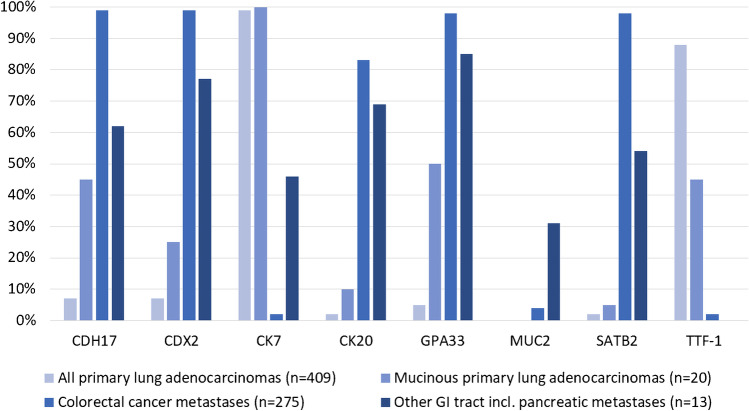
Frequency of positivity for selected immunostains in lung adenocarcinomas and pulmonary metastases from the gastrointestinal (GI) tract

The results of IHC staining for CDH17, GPA33, MUC2, MUC6, SATB2, and SMAD4, in primary non-squamous non-small-cell lung cancers and pulmonary metastases from the GI tract, are presented in Table 2 together with data for CDX2, CK7, CK20, and TTF-1 from our previous annotations for comparison [4, 13]. Representative images of positive staining in primary lung cancers and pulmonary metastases are found in Fig. 1. Further details for the primary lung cancers are found in Supplementary Table 1, also including data for squamous cell carcinomas (*n* = 186), carcinoid tumors (*n* = 7), and small-cell carcinomas (*n* = 3) in the cohorts. Correspondingly, further details for the pulmonary metastases are found in Supplementary Table 2, including data for various sites of origin for colorectal cancer metastases and data for other metastases including kidney cancers (*n* = 42), breast cancers (*n* = 27), gynecological (non-squamous) cancer (*n* = 17), prostatic cancer (*n* = 11), squamous cell carcinomas from various sites (*n* = 10), urothelial carcinomas (*n* = 8), adenoid cystic carcinomas (*n* = 6), thymomas (*n* = 5), hepatocellular carcinomas (*n* = 4), thyroid carcinomas (*n* = 3), and basal cell carcinoma (*n* = 1). Selected markers are also visualized in Fig. 2 for primary lung AC and pulmonary metastases from the GI tract.

**Table 2:** Frequency of positivity for immunostains in prevalent types of lung cancer and pulmonary metastases (see Supplementary Tables 1 and 2 for full data)

Pulmonary tumors	*n*	CDH17	CDX2	CK7	CK20	GPA33	MUC2	MUC6	SATB2	SMAD4^c^	TTF-1^c^
*Primary lung cancers*											
Adenocarcinoma^a^	409	29/409 (7%)	27/403 (7%)	403/408 (99%)	9/407 (2%)	19/408 (5%)	0/409 (0%)	19/409 (5%)	10/409 (2%)	42/407 (10%) / 11/407 (3%)	360/408 (88%)
Large-cell carcinoma	5	1/5 (20%)	3/5 (60%)	2/5 (40%)	0/5 (0%)	2/5 (40%)	0/5 (0%)	0/5 (0%)	2/5 (40%)	0/5 (0%) / 2/5 (40%)	0/5 (0%)
Sarcomatoid carcinoma	6	0/6 (0%)	0/6 (0%)	5/6 (83%)	0/6 (0%)	0/6 (0%)	0/6 (0%)	0/6 (0%)	0/6 (0%)	0/6 (0%) / 0/6 (0%)	3/6 (50%)
Large-cell neuroendocrine carcinoma	22	0/22 (0%)	5/22 (19%)	12/22 (55%)	0/22 (0%)	0/22 (0%)	0/22 (0%)	2/22 (9%)	10/22 (45%)	12/22 (55%) / 4/22 (18%)	16/22 (68%)
*Pulmonary metastases*											
Colorectal cancer	275	272/275 (99%)	271/273 (99%)	6/273 (2%)	228/274 (83%)	268/274 (98%)	10/274 (4%)	2/275 (0.7%)	269/275 (98%)	98/273 (36%) / 21/273 (8%)	5/275 (2%)
Pancreatic cancer	5	0/5 (0%)	2/5 (40%)	5/5 (100%)	2/5 (40%)	3/5 (60%)	0/5 (0%)	0/5 (0%)	0/5 (0%)	1/5 (20%) / 0/5 (0%)	0/5 (0%)
Other GI tract adenocarcinomas^b^	8	8/8 (100%)	8/8 (100%)	2/8 (25%)	6/8 (75%)	8/8 (100%)	4/8 (50%)	1/8 (13%)	6/7 (86%)	5/8 (63%) / 0/8 (0%)	0/8 (0%)

*CDH17*, cadherin 17; *CDX2*, caudal-type homeobox 2; *CK*, cytokeratin; *GI*, gastrointestinal; *GPA33*, glycoprotein A33; *MUC*, mucin; *SATB2*, special AT-rich sequence-binding protein 2; *SMAD4*, mothers against decapentaplegic homolog 4; *TTF-1*, thyroid transcription factor-1

^a^Including adenocarcinoma component of adenosquamous carcinomas and mixed large-cell neuroendocrine carcinoma and adenocarcinoma

^b^Four appendix, 2 small bowel (both ileal), 1 esophageal adenocarcinoma, and 1 cholangiocarcinoma

^c^Positive staining defined as ≥10% positive tumor cells except ≥1% for TTF-1 and preserved cytoplasmic only/preserved nuclear (w/wo cytoplasmic) for SMAD4

As evident from Table 2 and Supplementary Table 2, CDX2, CDH17, GPA33, and SATB2 exhibited very high and near-identical frequency of positivity in pulmonary metastases from colon (*n* = 113), rectum (*n* = 162), appendix (*n* = 4), small bowel (*n* = 2), cholangiocarcinoma (*n* = 1), and esophageal AC (*n* = 1). At least three of the markers were positive in all cases with two exceptions. Two metastases of rectal cancer from the same patient were negative for all four markers (and CK20 and TTF-1 while CK7 was positive). This unusual case has been previously described [38]. Also, CDX2 was not evaluable in one other metastasis of rectal cancer that was negative for SATB2.

Among non-GI non-squamous pulmonary metastases, described in Supplementary Table 2, the highest frequency for the markers was seen for CDX2 in gynecological cancers (41%; 7/17) and prostatic cancer (36%; 4/11), followed by CDH17 in gynecological cancers (24%; 4/17). SATB2 was infrequently positive in gynecological cancers (12%; 2/17) and kidney cancers (10%; 4/42), while GPA33-positivity was rare.

Table 2 and Supplementary Table 1 show that lung AC was infrequently positive for CDX2, CDH17, GPA33, and least commonly SATB2, while LCNEC was more often positive for SATB2.

CK20 was infrequently positive in lung AC (and negative in LCNEC) but slightly fewer metastases from the GI tract were positive for CK20 than for CDX2, CDH17, GPA33, and SATB2. MUC2 was negative in all primary lung cancers, but only positive in a limited number of pulmonary metastases including 10 colorectal cancers (whereof 8 were mucinous), 3 appendix AC, 1 cholangiocarcinoma, and 1 ductal breast cancer. The frequency of positivity for MUC6 and preserved SMAD4 was low and, in comparison, not that different between primary lung cancers and pulmonary metastases from the GI tract.

The frequency of positivity for the investigated markers in subgroups of lung AC based on mucinous (*n* = 20) or non-mucinous (*n* = 389) morphology and expression of TTF-1 as well as in CK7-positive pulmonary metastases with origin in the GI tract (*n* = 13), metastases of mucinous colorectal cancer (*n* = 17), and metastases of mucinous AC of other origins than colon/rectum (*n* = 6) are found in Table 3.

**Table 3:** Frequency of positivity for immunostains in subgroups of lung cancer and pulmonary metastases

Pulmonary tumors	*n*	CDH17	CDX2	CK7	CK20	GPA33	MUC2	MUC6	SATB2	SMAD4^d^	TTF-1^d^
*Primary lung cancers*											
Non-mucinous adenocarcinomas^a^	389	20/389 (5%)	22/383 (6%)	383/388 (99%)	7/387 (2%)	9/388 (2%)	0/389 (0%)	13/389 (3%)	9/389 (2%)	38/387 (10%) / 10/387 (3%)	351/388 (90%)
Whereof TTF-1 positive	351	14/351 (4%)	17/346 (5%)	349/351 (99%)	7/350 (2%)	7/350 (2%)	0/351 (0%)	12/351 (3%)	9/351 (3%)	35/349 (10%) / 9/349 (3%)	351/351 (100%)
Whereof TTF-1 negative	37	6/37 (16%)	5/36 (14%)	34/37 (92%)	0/37 (0%)	2/37 (5%)	0/37 (0%)	1/37 (3%)	0/37 (0%)	3/37 (8%) / 1/37 (3%)	0/37 (0%)
Mucinous adenocarcinomas	20	9/20 (45%)	5/20 (25%)	20/20 (100%)	2/20 (10%)	10/20 (50%)	0/20 (0%)	6/20 (30%)	1/20 (5%)	4/20 (20%) / 1/20 (5%)	9/20 (45%)
Whereof TTF-1 positive	9	3/9 (33%)	2/9 (22%)	9/9 (100%)	0/9 (0%)	4/9 (44%)	0/9 (0%)	1/9 (11%)	0/9 (0%)	2/9 (22%) / 0/9 (0%)	9/9 (100%)
Whereof TTF-1 negative	11	6/11 (55%)	3/11 (27%)	11/11 (100%)	2/11 (18%)	6/11 (55%)	0/11 (0%)	5/11 (45%)	1/11 (9%)	2/11 (18%) / 1/11 (9%)	0/9 (0%)
*Pulmonary metastases*											
CK7-positive GI tract adenocarcinoma^b^	13	6/13 (46%)	8/13 (62%)	13/13 (100%)	6/13 (46%)	8/13 (62%)	3/13 (23%)	1/13 (8%)	4/13 (31%)	5/13 (38%) / 0/13 (0%)	0/13 (0%)
Mucinous colorectal adenocarcinoma	17	17/17 (100%)	16/16 (100%)	2/16 (13%)	16/17 (94%)	13/16 (81%)	8/16 (50%)	0/17 (0%)	17/17 (100%)	3/16 (19%) / 4/16 (25%)	0/17 (0%)
Other mucinous adenocarcinoma^c^	6	4/6 (67%)	5/5 (100%)	3/6 (50%)	3/6 (50%)	2/6 (33%)	2/6 (33%)	1/6 (17%)	1/5 (20%)	4/6 (67%) / 1/6 (17%)	0/6 (0%)

*CDH17*, cadherin 17; *CDX2*, caudal-type homeobox 2; *CK*, cytokeratin; *GI*, gastrointestinal; *GPA33*, glycoprotein A33; *MUC*, mucin; *SATB2*, special AT-rich sequence-binding protein 2; *SMAD4*, mothers against decapentaplegic homolog 4; *TTF-1*, thyroid transcription factor-1

^a^Including adenocarcinoma component of adenosquamous carcinomas and mixed large-cell neuroendocrine carcinoma and adenocarcinoma

^b^Five from rectum, 5 from pancreas, and 1 each from right colon, esophagus, and cholangiocarcinoma

^c^Two from appendix and 1 each from breast, ovarium, pancreas, and vulva

^d^Positive staining defined as ≥10% positive tumor cells except ≥1% for TTF-1 and preserved cytoplasmic only/preserved nuclear (with/without cytoplasmic) for SMAD4

As evident, especially CDH17, CDX2, CK20, GPA33, and MUC6 were more frequently positive in mucinous than non-mucinous lung AC, while SATB2 positivity was uncommon. Also, CDH17 and CDX2 showed a slightly higher frequency of expression in TTF-1-negative than TTF-1-positive cases for both non-mucinous and mucinous AC. Two of the three TTF-1- and CK7-negative lung AC were poorly differentiated with solid growth while one was acinar predominant (and partly positive for TTF-1 clone SPT24; data not shown).

CDX2 was the most sensitive marker for pulmonary metastases of mucinous AC from other sites, followed by CDH17. MUC2 was positive in slightly less than half of metastatic mucinous AC, but also negative in all primary mucinous AC. CDX2 and GPA33 were the most sensitive GI markers in the CK7-positive AC metastases with origin in the GI tract, while only a few cases were positive for MUC2. See Table 3.

**Fig. 3:**
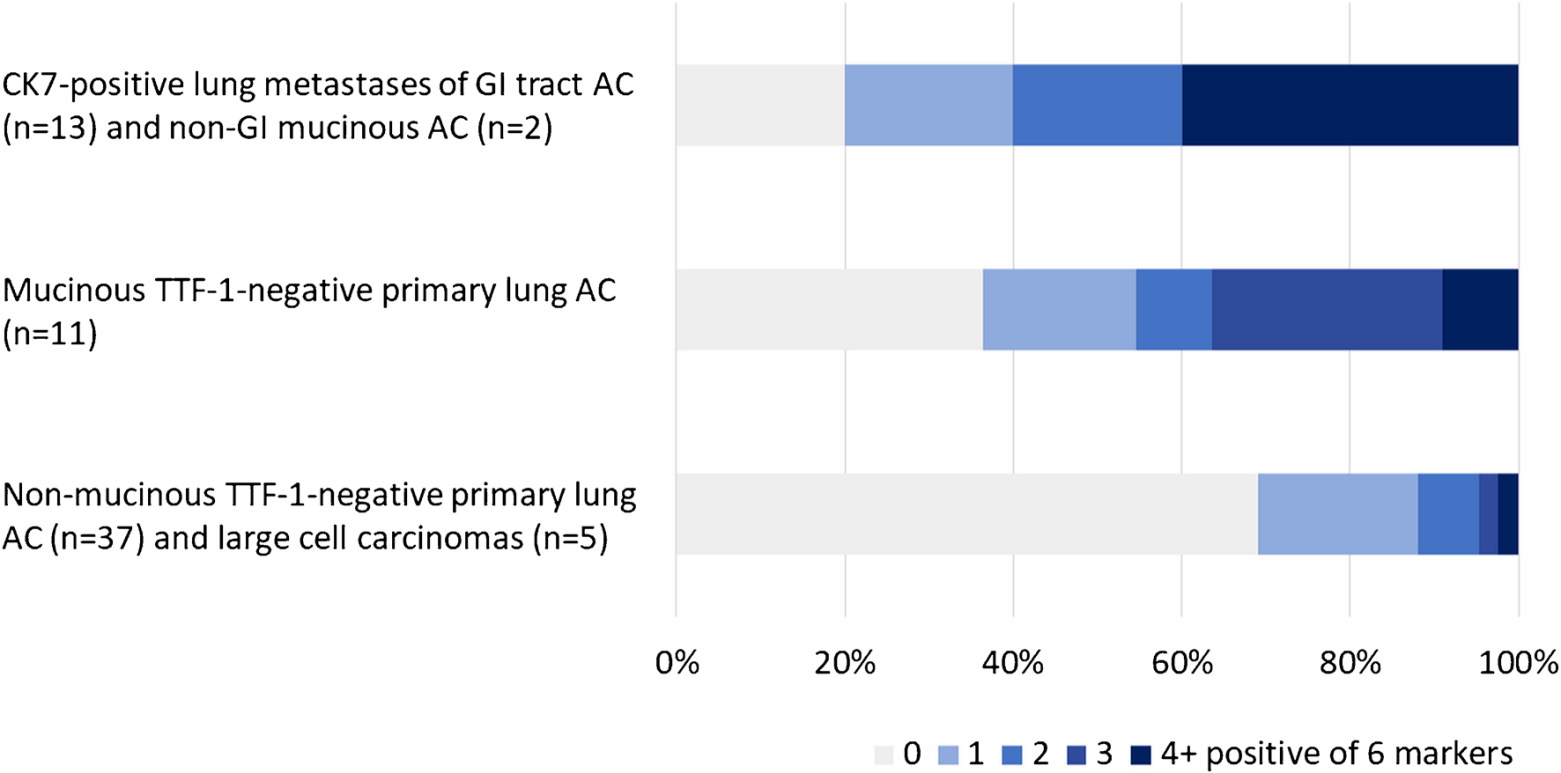
Number of positive immunostains (≥10% positive tumor cells) of the six gastrointestinal (GI) markers CDH17, CDX2, CK20, GPA33, MUC2, and SATB2 in subgroups of lung cancer and pulmonary adenocarcinoma (AC) metastases

Co-occurrence of expression of the GI markers CDH17, CDX2, CK20, GPA33, MUC2, and SATB2 (i.e., MUC6 and SMAD4 excluded of the investigated markers) for subgroups of lung cancer and pulmonary metastases are presented in Table 4 and Fig. 3. All primary lung sarcomatoid carcinomas were negative for all markers (not included in the table and figure). The two metastases of CK7-positive AC from the GI tract that were negative for all markers were the two rectal cancer metastases from the same patient described above, while the metastasis of non-colorectal mucinous AC that was negative for all markers was the mucinous breast cancer (CDX2 was not evaluable for that case). All TTF-1-positive metastases of colorectal cancer were positive for five of the GI markers (not included in Table 4 since not mucinous or positive for CK7). Non-GI pulmonary metastases with two positive GI markers included four gynecological cancers, one kidney cancer (papillary, also CK7-positive), one prostatic cancer, and one urothelial carcinoma (also CK7-positive). One other gynecological cancer was the only non-GI metastasis with three positive markers.

**Table 4:** Number of positive immunostains (≥10% positive tumor cells) of the six gastrointestinal markers CDH17, CDX2, CK20, GPA33, MUC2, and SATB2 in subgroups of lung cancer and pulmonary metastases

Pulmonary tumors	*n*	0	1	2	3	4+
*Primary lung cancers*						
Non-mucinous TTF-1-positive adenocarcinoma	351	309/351 (88%)	34/351 (10%)	5/351 (1%)	3/351 (1%)	0/351 (0%)
Non-mucinous TTF-1-negative adenocarcinoma	37	28/37 (76%)	6/37 (16%)	2/37 (5%)	1/37 (3%)	0/37 (0%)
Mucinous TTF-1-positive adenocarcinoma	9	4/9 (44%)	2/9 (22%)	2/9 (22%)	1/9 (11%)	0/9 (0%)
Mucinous TTF-1-negative adenocarcinoma	11	4/11 (36%)	2/11 (18%)	1/11 (9%)	3/11 (27%)	1/11 (9%)
Large-cell carcinoma	5	1/5 (20%)	2/5 (40%)	1/5 (20%)	0/5 (0%)	1/5 (20%)
Large-cell neuroendocrine carcinoma	22	9/22 (41%)	12/22 (55%)	1/22 (5%)	0/22 (0%)	0/22 (0%)
*Pulmonary metastases*						
CK7-positive GI tract adenocarcinoma^a^	13	2/13 (15%)	3/13 (23%)	2/13 (15%)	0/13 (0%)	6/13 (46%)
Mucinous colorectal adenocarcinoma	17	0/17 (0%)	0/17 (0%)	0/17 (0%)	0/17 (0%)	17/17 (100%)
Other mucinous adenocarcinoma^b^	6	1/6 (17%)	1/6 (17%)	1/6 (17%)	1/6 (17%)	2/6 (33%)

*CDH17*, cadherin 17; *CDX2*, caudal-type homeobox 2; *CK*, cytokeratin; *GI*, gastrointestinal; *GPA33*, glycoprotein A33; *MUC*, mucin; *SATB2*, special AT-rich sequence-binding protein 2; *TTF-1*, thyroid transcription factor-1

^a^Five from rectum, 5 from pancreas, and 1 each from right colon, esophagus, and cholangiocarcinoma

^e^Two from appendix and 1 each from breast (CDX2 not evaluable, negative for all other markers), ovarium, pancreas, and vulva

## Discussion

In the present study, we investigated if there may be alternatives to the panel CDX2, CK7, CK20, and TTF-1 for differentiation between primary lung cancer and pulmonary metastases from the GI tract. As also previously known [39–44], if a pulmonary tumor is negative for CK7, it is rarely primary lung AC, and if positive for TTF-1 (clone 8G7G3/1), it is rarely metastatic AC to the lung. Our data further support that CDH17, GPA33, and SATB2 in addition to CDX2 are all very sensitive markers for colorectal cancer metastases, and slightly more sensitive than CK20. Although evaluated in a limited number of cases, the markers also exhibited a good diagnostic value for other GI tract origin, especially CDH17 and GPA33 in addition to CDX2. However, the sensitivity was only moderate for GPA33 (as for CDX2 and CK20), and low for CDH17 and SATB2, for pancreatic origin, but the limited number of pulmonary metastases from the pancreas in our material prevent any strong conclusions.

In the literature, the sensitivity for CDH17 has been reported to be 96–100% for colorectal, 18–57% for pancreatic, 25–90% for gastric, and 39–82% for esophageal AC, and often higher than CDX2 for the upper GI tumors [15–18]. Correspondingly, a sensitivity of 95–96% for colorectal, 4–50% for pancreatic, and 58% for gastric AC have been reported for GPA33 [19, 20]. The broad range of sensitivity and the limited number of studies with large upper GI tumor cohorts call for further investigation. More cases have been evaluated for SATB2, with a reported sensitivity of 80–100% for colorectal, 0–5% for pancreatic, 0–19% for gastric, and 7–12% for esophageal AC [21–24]. Still, only a few studies include metastases of GI tumors to the lungs and comparisons of all the markers in the same material are missing. One limited study on enteric lung AC and metastatic colorectal cancer presented a diagnostic gain of CDH17 and SATB2 [45], while another study on cytological samples from various sites did not find any benefit in adding the two markers [46].

In our investigation, MUC2 was the most specific marker but with a very limited sensitivity for GI tract metastases, in line with the literature [8, 25–27], while MUC6 and SMAD4 did not contribute to differential diagnostics. However, despite several protocol optimization efforts, the staining for SMAD4 was rather weak (see Supplementary Figure 1 for staining of control tissue). Thus, the conclusions for SMAD4 should be interpreted with care.

There was no single marker or panel to perfectly separate lung AC from pulmonary metastases from the GI tract. Based on our data, the best panel, in addition to CK7 and TTF-1, may be CDX2 and/or GPA33 plus MUC2 for a combination of sensitive and specific markers, or all CDH17, CDX2, CK20, GPA33, MUC2, and SATB2 with four positive GI markers supporting metastasis. Investigations including biopsies and cytological samples from the lungs are needed to further evaluate these two alternatives and their value in the clinical setting. Mucinous AC is the diagnostically most challenging group of primary lung cancers, and mainly data on CDX2 and CK20 is found in the literature, with 0–10% and 31–60% positive cases, respectively, in studies reporting both markers [10, 12, 47]. Given our findings, with a higher frequency of CDX2 than CK20 and especially prevalent GPA33 and CDH17 expression, further studies of this tumor subtype would be of value.

Non-mucinous pulmonary metastases from non-GI organs are often diagnostically less problematic. However, the expression of GI markers in metastases from gynecological cancers was notable in our material. Three gynecological cancers expressing at least one GI marker were negative for PAX8 (data not shown), including two cervical AC and one mucinous AC of the vulva (the latter included in Tables 3 and 4). Expression of GI markers especially in mucinous gynecological AC is well known [48]. In the clinical diagnostic situation, data on non-GI metastases may be of interest, e.g., for cancer of unknown primary with involvement of the lungs, especially since broad IHC panels are often applied in these situations.

Interestingly, primary pulmonary TTF-1-negative non-mucinous AC as well as large-cell carcinomas expressed GI markers more often than TTF-1-positive non-mucinous AC in our material. Still, very few such cases expressed more than two markers. Also, LCNEC expressed GI markers more often than small-cell carcinomas and carcinoids (Supplementary Table 1). Based on multidisciplinary information, there was no suspicion from the clinical setting that any of the primary lung cancers in our cohorts were GI metastases.

The main strengths of our study include the use of well-investigated primary lung cancers and pulmonary metastases with a large number of colorectal cancer metastases, and that multiple markers were compared in the same large material. Also, we present data on relevant subgroups such as TTF-1-negative lung AC, CK7-positive metastases of GI AC, and mucinous AC. There are some limitations to the study. Most importantly, the cohorts included a limited number (*n* = 7) of metastases from the upper GI tract. We chose not to include primary upper GI tract AC, as a population of primary tumors from other sites may differ from lung metastases, which is the clinically relevant type and study’s focus. Also, our material did not include bronchial or lung biopsies, which are relevant, but often contain limited material for extensive investigations and where the diagnosis often relies on the evaluated IHC markers. Furthermore, while TMAs are not optimal for predicting the exact frequency of positivity in resected material or biopsies, it is a very good material for marker comparison since the same area is investigated for many cases.

In conclusion, this comprehensive comparison supports that CDH17, GPA33, and SATB2 may be used as equivalent alternatives to CDX2 and CK20. Also, MUC2 is a specific marker but exhibits low sensitivity, and a combination of several markers may be considered to support GI tract origin in selected cases of pulmonary tumors. However, there is no single marker or panel of markers to distinguish primary lung cancers from GI tract or mucinous metastases with certainty.

### Supplementary information

ESM 1

## Data Availability

The data that support the findings of this study are available from the corresponding author, H. B., upon reasonable request.
