# Supplementary material for: Diagnostic gastrointestinal markers in primary lung cancer and pulmonary metastases
Source: Virchows Arch. 2023 Jun 22;485(2):347–57. doi: 10.1007/s00428-023-03583-w (PMC11329406; doi:10.1007/s00428-023-03583-w)
Supplement: Supplementary file 1 — ESM 1 [file 428_2023_3583_MOESM1_ESM.zip › 428_2023_3583_MOESM1_ESM/Suppl figure 1.pdf]

Supplementary Figure 1. Positive control tissue (scale bar is 0.1 mm).

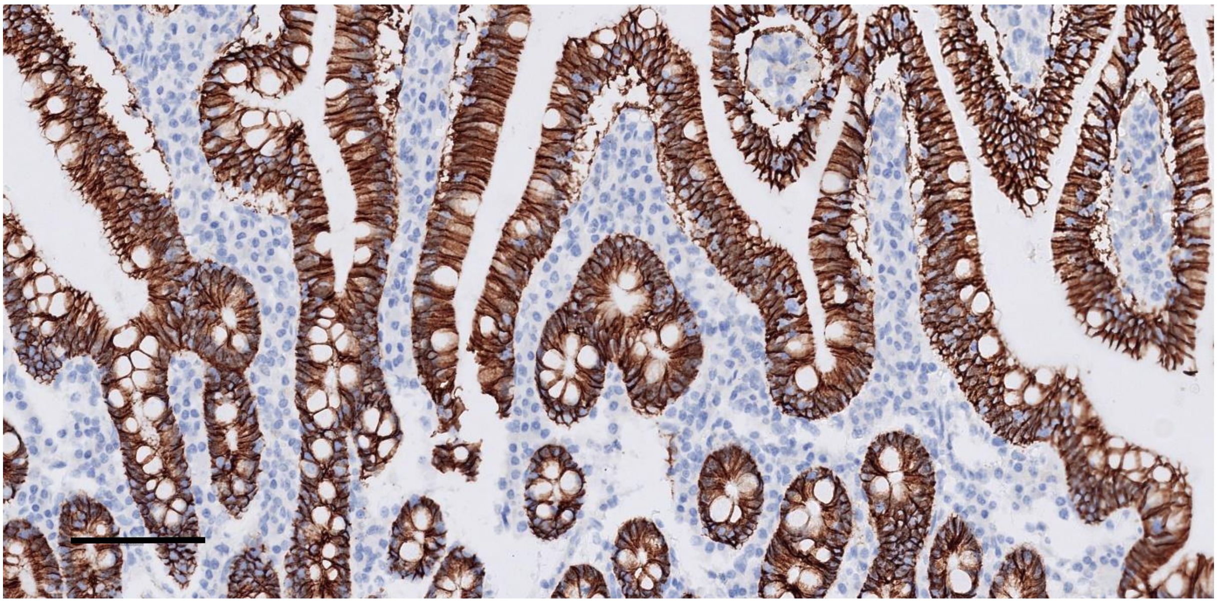

A. CDH17, small intestine.

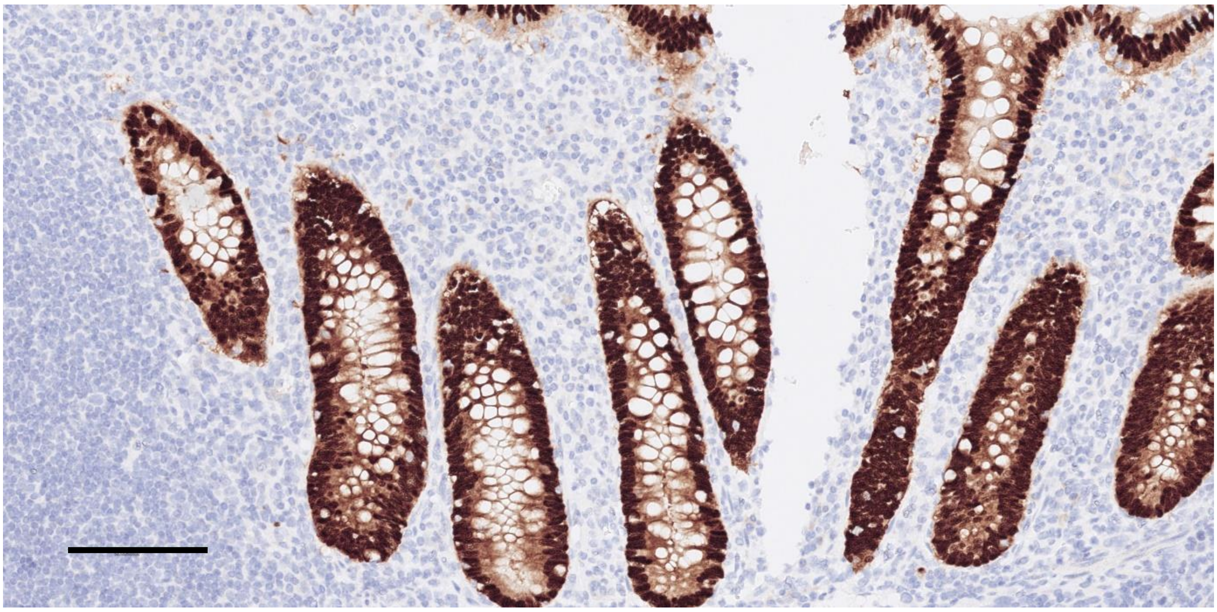

B. CDX2, appendix.

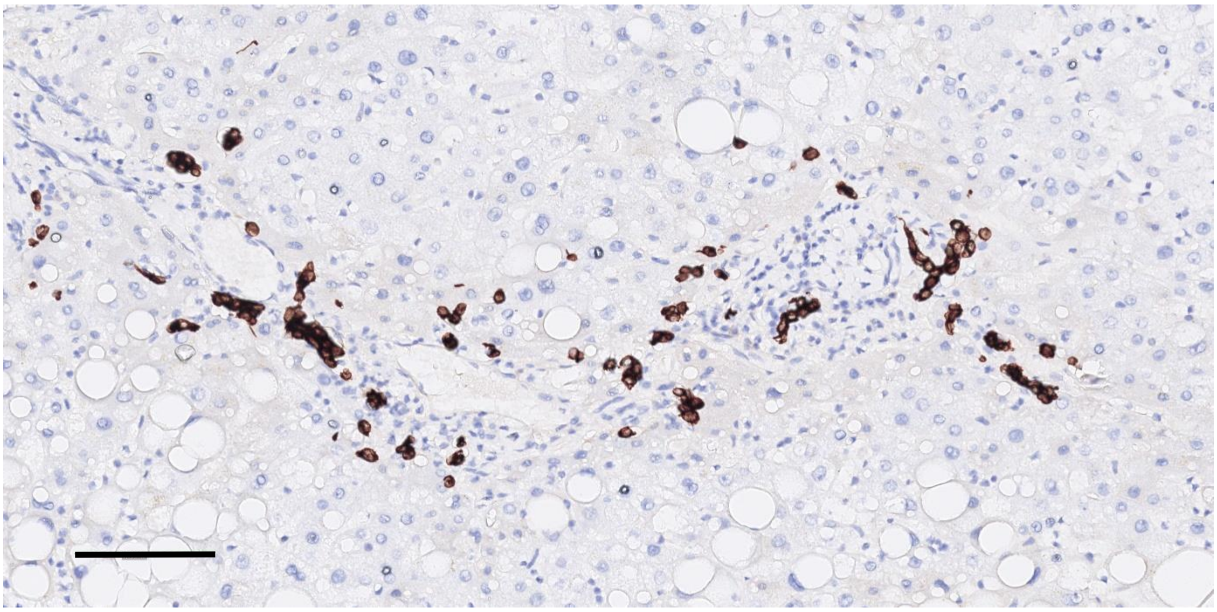

C. CK7, liver.

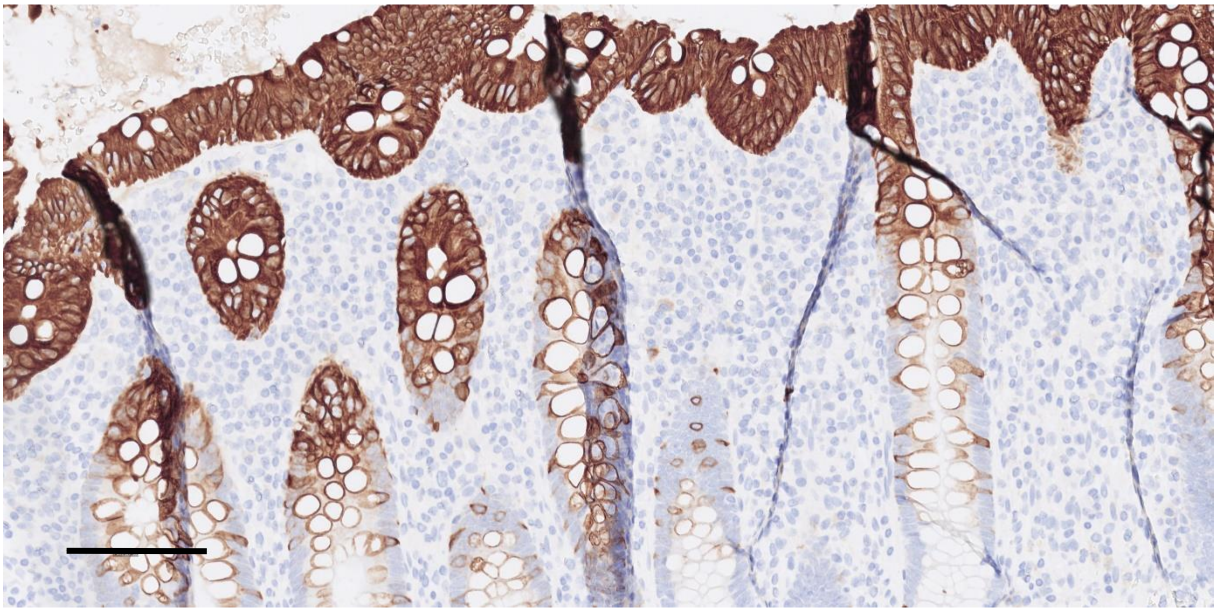

D. CK20, appendix.

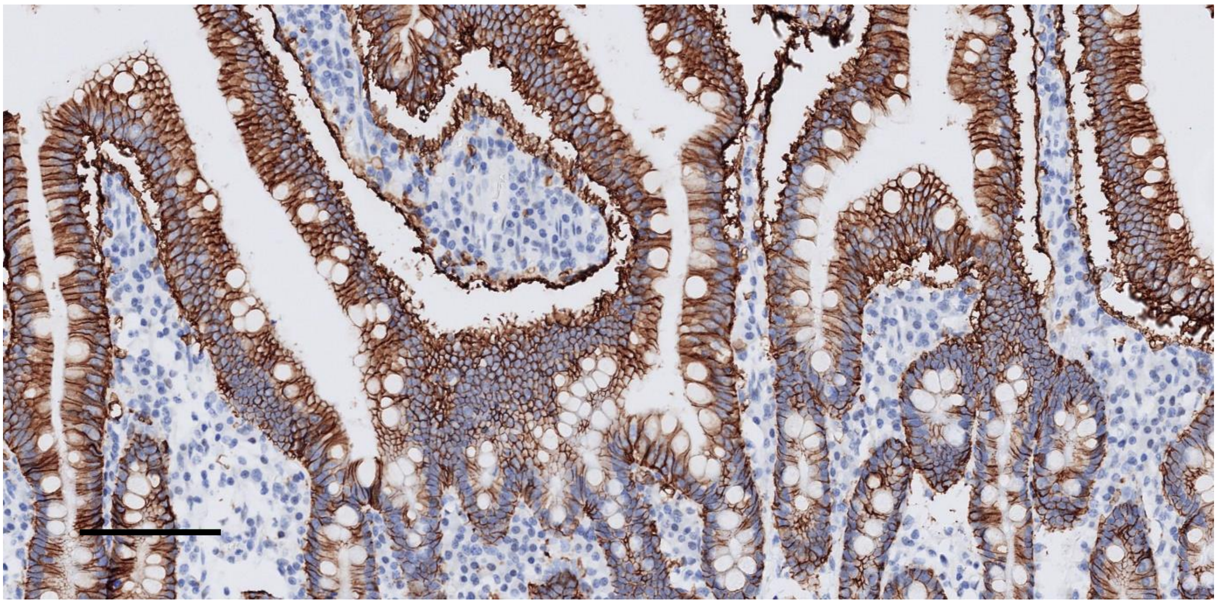

E. GPA33, small intestine.

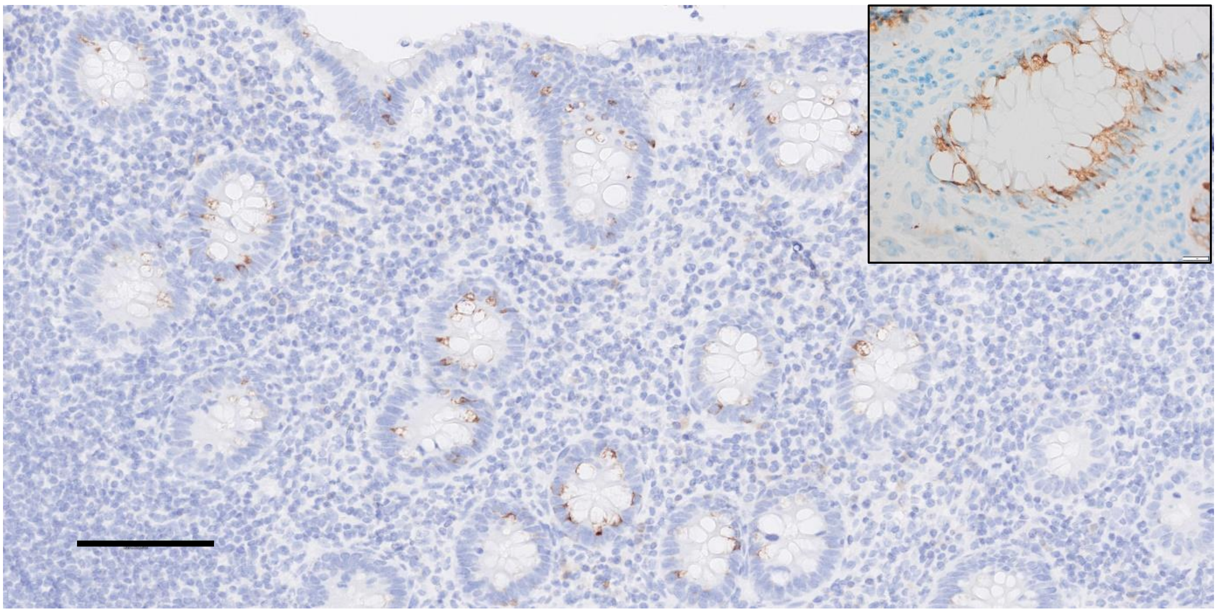

F. MUC2, appendix. Inset is colon as internal control (from primary colorectal cancer in the TMAs).

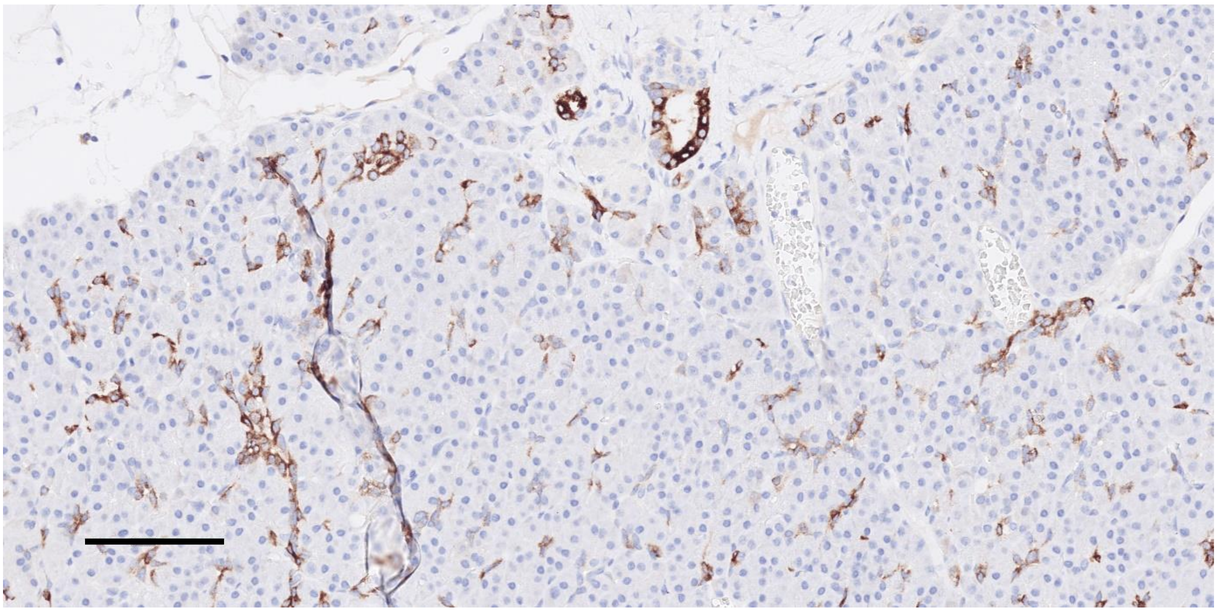

G. MUC6, pancreas.

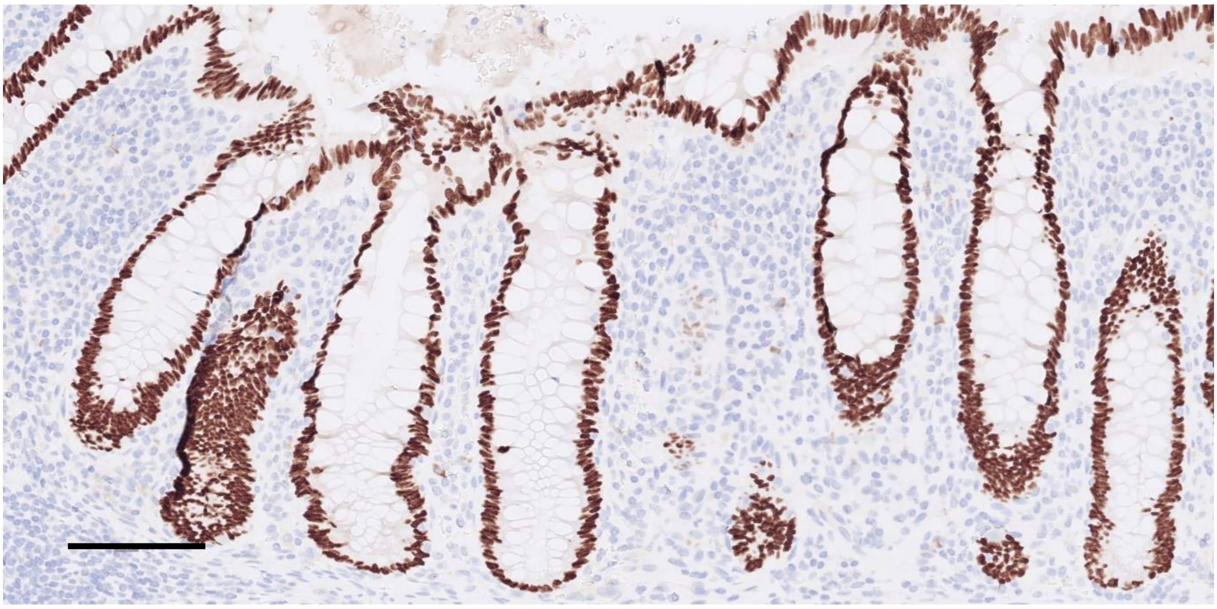

H. SATB2, appendix.

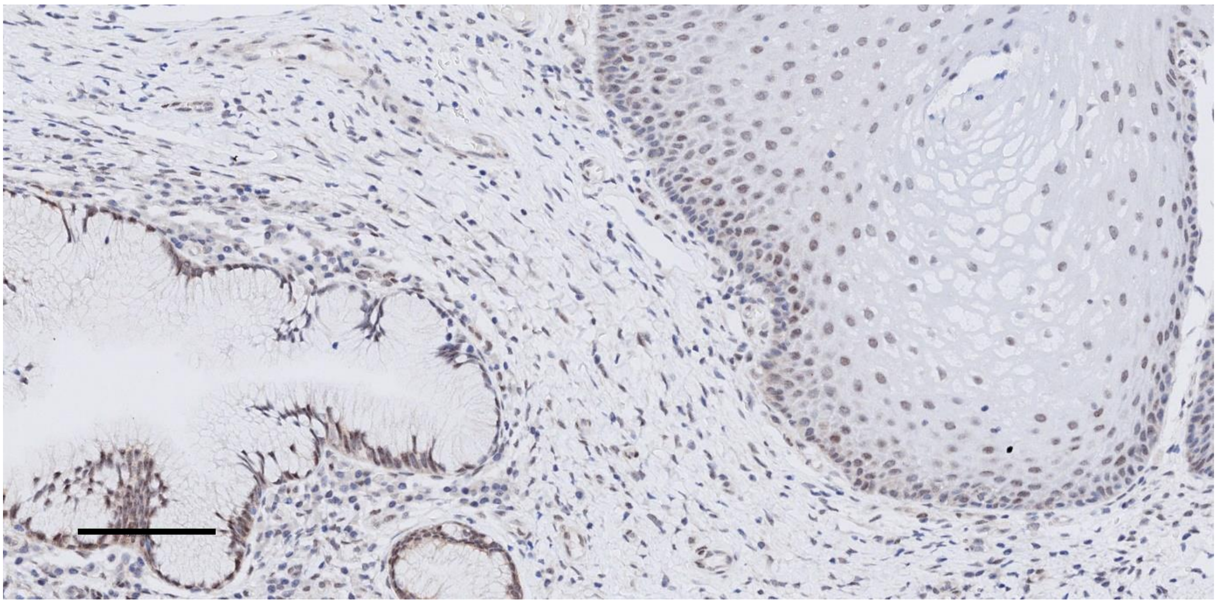

I. SMAD4, cervix.

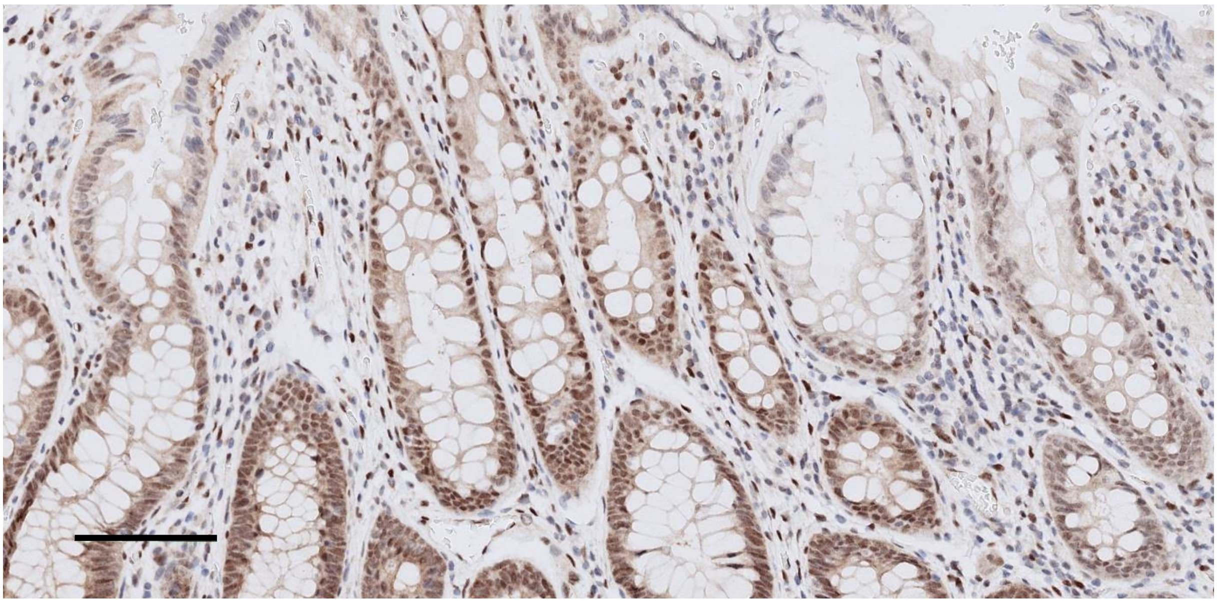

J, SMAD4, colon.

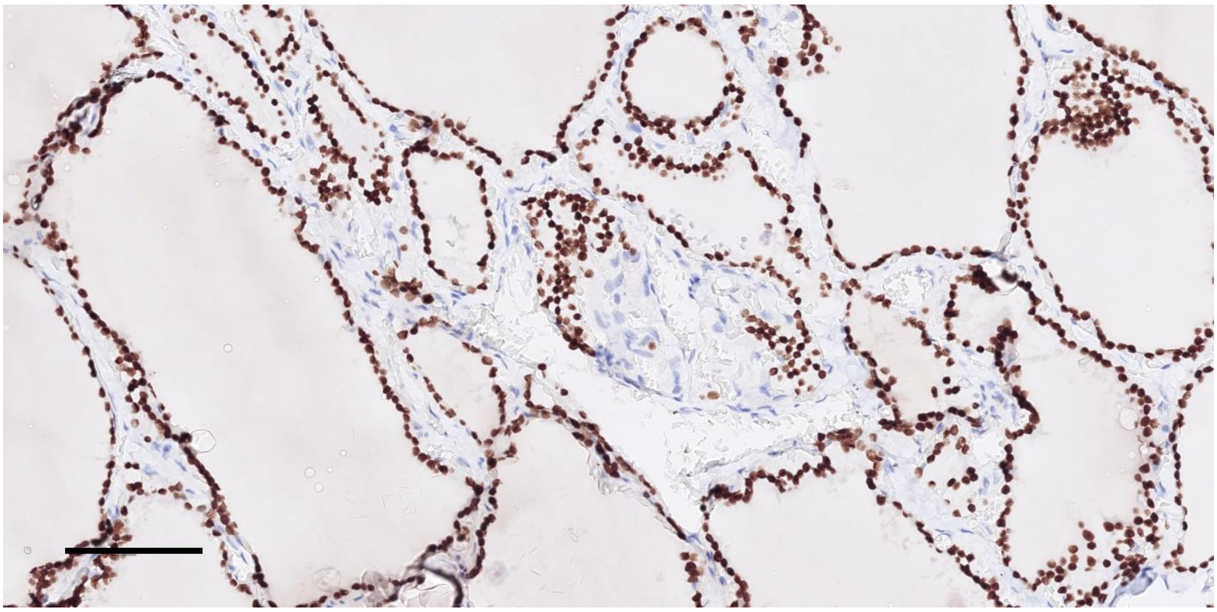

K. TTF-1, thyroid.
