# Supplementary material for: Diagnostic gastrointestinal markers in primary lung cancer and pulmonary metastases
Source: Virchows Arch. 2023 Jun 22;485(2):347–57. doi: 10.1007/s00428-023-03583-w (PMC11329406; doi:10.1007/s00428-023-03583-w)
Supplement: Supplementary file 1 — ESM 1 [file 428_2023_3583_MOESM1_ESM.zip › 428_2023_3583_MOESM1_ESM/Suppl table 1.docx]

**Supplementary Table 1.** Results of immunohistochemical staining of tissue microarrays with resected primary lung cancers, with stratification based on percent positive tumor cells.

| **Tumor type** | **No. of tumors** | **CDH17** | **CDX2** | **CK7** | **CK20** | **GPA33** | **MUC2** | **MUC6** | **SATB2** | **TTF-1** |
| --- | --- | --- | --- | --- | --- | --- | --- | --- | --- | --- |
| **Adenocarcinoma ^a^** | **409** |  |  |  |  |  |  |  |  |  |
| <1% |  | 372 | 358 | 5 | 388 | 385 | 409 | 382 | 389 | 48 |
| 1-9% |  | 8 | 18 | 0 | 10 | 4 | 0 | 8 | 10 | 17 |
| 10-24% |  | 11 | 8 | 2 | 0 | 9 | 0 | 10 | 7 | 11 |
| 25-49% |  | 10 | 9 | 5 | 5 | 5 | 0 | 3 | 3 | 30 |
| 50%+ |  | 8 | 10 | 396 | 4 | 5 | 0 | 6 | 0 | 302 |
| Missing |  | 0 | 6 | 1 | 2 | 1 | 0 | 0 | 0 | 1 |
| ***- whereof mucinous adenocarcinoma*** | ***20*** |  |  |  |  |  |  |  |  |  |
| *<1%* |  | *10* | *12* | *0* | *14* | *10* | *20* | *11* | *19* | *11* |
| *1-9%* |  | *1* | *3* | *0* | *4* | *0* | *0* | *3* | *0* | *2* |
| *10-24%* |  | *3* | *3* | *0* | *0* | *6* | *0* | *5* | *1* | *1* |
| *25-49%* |  | *3* | *0* | *2* | *0* | *1* | *0* | *0* | *0* | *0* |
| *50%+* |  | *3* | *2* | *18* | *2* | *3* | *0* | *1* | *0* | *6* |
| *Missing* |  | *0* | *0* | *0* | *0* | *0* | *0* | *0* | *0* | *0* |
| **Squamous cell carcinoma ^b^** | **186** |  |  |  |  |  |  |  |  |  |
| <1% |  | 186 | 159 | 102 | 182 | 186 | 186 | 184 | 181 | 186 |
| 1-9% |  | 0 | 12 | 5 | 2 | 0 | 0 | 1 | 3 | 0 |
| 10-24% |  | 0 | 3 | 8 | 1 | 0 | 0 | 0 | 1 | 0 |
| 25-49% |  | 0 | 2 | 10 | 0 | 0 | 0 | 0 | 0 | 0 |
| 50%+ |  | 0 | 8 | 61 | 1 | 0 | 0 | 0 | 0 | 0 |
| Missing |  | 0 | 2 | 0 | 0 | 0 | 0 | 1 | 1 | 0 |
| **Large cell carcinoma** | **5** |  |  |  |  |  |  |  |  |  |
| <1% |  | 4 | 2 | 3 | 5 | 3 | 5 | 5 | 3 | 5 |
| 1-9% |  | 0 | 0 | 0 | 0 | 0 | 0 | 0 | 0 | 0 |
| 10-24% |  | 0 | 1 | 0 | 0 | 1 | 0 | 0 | 1 | 0 |
| 25-49% |  | 0 | 0 | 0 | 0 | 1 | 0 | 0 | 0 | 0 |
| 50%+ |  | 1 | 2 | 2 | 0 | 0 | 0 | 0 | 1 | 0 |
| Missing |  | 0 | 0 | 0 | 0 | 0 | 0 | 0 | 0 | 0 |
| **Sarcomatoid carcinoma ^c^** | **6** |  |  |  |  |  |  |  |  |  |
| <1% |  | 6 | 6 | 1 | 6 | 6 | 6 | 5 | 6 | 3 |
| 1-9% |  | 0 | 0 | 0 | 0 | 0 | 0 | 1 | 0 | 1 |
| 10-24% |  | 0 | 0 | 0 | 0 | 0 | 0 | 0 | 0 | 0 |
| 25-49% |  | 0 | 0 | 0 | 0 | 0 | 0 | 0 | 0 | 0 |
| 50%+ |  | 0 | 0 | 5 | 0 | 0 | 0 | 0 | 0 | 2 |
| Missing |  | 0 | 0 | 0 | 0 | 0 | 0 | 0 | 0 | 0 |
| **Small cell carcinoma** | **3** |  |  |  |  |  |  |  |  |  |
| <1% |  | 3 | 3 | 2 | 3 | 3 | 3 | 3 | 1 | 2 |
| 1-9% |  | 0 | 0 | 0 | 0 | 0 | 0 | 0 | 1 | 0 |
| 10-24% |  | 0 | 0 | 0 | 0 | 0 | 0 | 0 | 1 | 0 |
| 25-49% |  | 0 | 0 | 0 | 0 | 0 | 0 | 0 | 0 | 0 |
| 50%+ |  | 0 | 0 | 1 | 0 | 0 | 0 | 0 | 0 | 1 |
| Missing |  | 0 | 0 | 0 | 0 | 0 | 0 | 0 | 0 | 0 |
| **Large cell neuroendocrine carcinoma** | **22** |  |  |  |  |  |  |  |  |  |
| <1% |  | 22 | 14 | 9 | 22 | 22 | 22 | 17 | 10 | 6 |
| 1-9% |  | 0 | 3 | 1 | 0 | 0 | 0 | 3 | 2 | 1 |
| 10-24% |  | 0 | 2 | 1 | 0 | 0 | 0 | 1 | 1 | 0 |
| 25-49% |  | 0 | 1 | 0 | 0 | 0 | 0 | 1 | 2 | 2 |
| 50%+ |  | 0 | 1 | 11 | 0 | 0 | 0 | 0 | 7 | 13 |
| Missing |  | 0 | 1 | 0 | 0 | 0 | 0 | 0 | 0 | 0 |
| **Carcinoid tumor** | **7** |  |  |  |  |  |  |  |  |  |
| <1% |  | 6 | 7 | 1 | 7 | 7 | 7 | 7 | 7 | 3 |
| 1-9% |  | 0 | 0 | 2 | 0 | 0 | 0 | 0 | 0 | 3 |
| 10-24% |  | 0 | 0 | 0 | 0 | 0 | 0 | 0 | 0 | 0 |
| 25-49% |  | 1 | 0 | 0 | 0 | 0 | 0 | 0 | 0 | 1 |
| 50%+ |  | 0 | 0 | 4 | 0 | 0 | 0 | 0 | 0 | 0 |
| Missing |  | 0 | 0 | 0 | 0 | 0 | 0 | 0 | 0 | 0 |

Abbreviations: CDH17, cadherin 17; CDX2, caudal type homeobox 2; CK, cytokeratin; GPA33, glycoprotein A33; MUC, mucin; SATB2, special AT-rich sequence-binding protein 2; TTF-1, thyroid transcription factor-1

^a^ Including the adenocarcinoma component of 8 adenosquamous carcinomas and 4 combined large cell neuroendocrine carcinomas

^b^ Including the squamous cell carcinoma component of 5 adenosquamous carcinomas

^c^ 5 pleomorphic carcinomas with an adenocarcinoma component and 1 giant cell carcinoma
