# Supplementary material for: Diagnostic gastrointestinal markers in primary lung cancer and pulmonary metastases
Source: Virchows Arch. 2023 Jun 22;485(2):347–57. doi: 10.1007/s00428-023-03583-w (PMC11329406; doi:10.1007/s00428-023-03583-w)
Supplement: Supplementary file 1 — ESM 1 [file 428_2023_3583_MOESM1_ESM.zip › 428_2023_3583_MOESM1_ESM/Suppl table 2.docx]

**Supplementary Table 2.** Results of immunohistochemical staining of tissue microarrays with resected epithelial pulmonary metastases, with stratification based on percent positive tumor cells.

| **Tumor origin or type** | **No. of tumors** | **CDH17** | **CDX2** | **CK7** | **CK20** | **GPA33** | **MUC2** | **MUC6** | **SATB2** | **TTF-1** |
| --- | --- | --- | --- | --- | --- | --- | --- | --- | --- | --- |
| **Colorectal carcinoma ^a^** | 275 |  |  |  |  |  |  |  |  |  |
| <1% |  | 3 | 2 | 265 | 26 | 3 | 252 | 267 | 5 | 270 |
| 1-9% |  | 0 | 0 | 2 | 20 | 3 | 12 | 6 | 1 | 0 |
| 10-24% |  | 1 | 0 | 2 | 22 | 6 | 5 | 1 | 3 | 2 |
| 25-49% |  | 2 | 0 | 1 | 43 | 3 | 4 | 0 | 12 | 2 |
| 50%+ |  | 269 | 271 | 3 | 163 | 259 | 1 | 1 | 254 | 1 |
| Missing |  | 0 | 2 | 2 | 1 | 1 | 1 | 0 | 0 | 0 |
| ***- whereof origin right colon*** | *21* |  |  |  |  |  |  |  |  |  |
| *<1%* |  | *0* | *0* | *19* | *1* | *0* | *20* | *19* | *1* | *21* |
| *1-9%* |  | *0* | *0* | *0* | *1* | *1* | *0* | *2* | *0* | *0* |
| *10-24%* |  | *0* | *0* | *1* | *3* | *3* | *1* | *0* | *0* | *0* |
| *25-49%* |  | *0* | *0* | *0* | *2* | *0* | *0* | *0* | *1* | *0* |
| *50%+* |  | *21* | *21* | *0* | *14* | *17* | *0* | *0* | *19* | *0* |
| *Missing* |  | *0* | *0* | *1* | *0* | *0* | *0* | *0* | *0* | *0* |
| ***- whereof origin left colon*** | *92* |  |  |  |  |  |  |  |  |  |
| *<1%* |  | *0* | *0* | *91* | *6* | *1* | *86* | *90* | *1* | *91* |
| *1-9%* |  | *0* | *0* | *1* | *8* | *0* | *4* | *2* | *0* | *0* |
| *10-24%* |  | *0* | *0* | *0* | *10* | *0* | *1* | *0* | *0* | *0* |
| *25-49%* |  | *0* | *0* | *0* | *13* | *3* | *1* | *0* | *3* | *0* |
| *50%+* |  | *92* | *92* | *0* | *55* | *88* | *0* | *0* | *88* | *1* |
| *Missing* |  | *0* | *0* | *0* | *0* | *0* | *0* | *0* | *0* | *0* |
| ***- whereof origin rectum*** | *162* |  |  |  |  |  |  |  |  |  |
| *<1%* |  | *3* | *2* | *155* | *19* | *2* | *146* | *158* | *3* | *158* |
| *1-9%* |  | *0* | *0* | *1* | *11* | *2* | *8* | *2* | *1* | *0* |
| *10-24%* |  | *1* | *0* | *1* | *9* | *3* | *3* | *1* | *3* | *2* |
| *25-49%* |  | *2* | *0* | *1* | *28* | *0* | *3* | *0* | *8* | *2* |
| *50%+* |  | *156* | *158* | *3* | *94* | *154* | *1* | *1* | *147* | *0* |
| *Missing* |  | *0* | *2* | *1* | *1* | *1* | *1* | *0* | *0* | *0* |
| ***- whereof mucinous (any origin)*** | *17* |  |  |  |  |  |  |  |  |  |
| *<1%* |  | *0* | *0* | *14* | *1* | *0* | *7* | *17* | *0* | *17* |
| *1-9%* |  | *0* | *0* | *0* | *0* | *3* | *1* | *0* | *0* | *0* |
| *10-24%* |  | *0* | *0* | *1* | *0* | *1* | *5* | *0* | *1* | *0* |
| *25-49%* |  | *1* | *0* | *1* | *2* | *0* | *2* | *0* | *3* | *0* |
| *50%+* |  | *16* | *16* | *0* | *14* | *12* | *1* | *0* | *13* | *0* |
| *Missing* |  | *0* | *1* | *1* | *0* | *1* | *1* | *0* | *0* | *0* |
| **Appendix carcinoma** | 4 |  |  |  |  |  |  |  |  |  |
| <1% |  | 0 | 0 | 4 | 0 | 0 | 0 | 4 | 0 | 4 |
| 1-9% |  | 0 | 0 | 0 | 0 | 0 | 1 | 0 | 0 | 0 |
| 10-24% |  | 1 | 0 | 0 | 0 | 0 | 1 | 0 | 0 | 0 |
| 25-49% |  | 0 | 0 | 0 | 0 | 1 | 1 | 0 | 1 | 0 |
| 50%+ |  | 3 | 4 | 0 | 4 | 3 | 1 | 0 | 2 | 0 |
| Missing |  | 0 | 0 | 0 | 0 | 0 | 0 | 0 | 1 | 0 |
| **Small bowel (ilieal) carcinoma** | 2 |  |  |  |  |  |  |  |  |  |
| <1% |  | 0 | 0 | 2 | 0 | 0 | 1 | 2 | 0 | 2 |
| 1-9% |  | 0 | 0 | 0 | 0 | 0 | 1 | 0 | 0 | 0 |
| 10-24% |  | 0 | 0 | 0 | 0 | 1 | 0 | 0 | 1 | 0 |
| 25-49% |  | 0 | 0 | 0 | 0 | 0 | 0 | 0 | 0 | 0 |
| 50%+ |  | 2 | 2 | 0 | 2 | 1 | 0 | 0 | 1 | 0 |
| Missing |  | 0 | 0 | 0 | 0 | 0 | 0 | 0 | 0 | 0 |
| **Esophageal adenocarcinoma** | 1 |  |  |  |  |  |  |  |  |  |
| <1% |  | 0 | 0 | 0 | 0 | 0 | 1 | 0 | 0 | 1 |
| 1-9% |  | 0 | 0 | 0 | 1 | 0 | 0 | 0 | 0 | 0 |
| 10-24% |  | 0 | 0 | 0 | 0 | 0 | 0 | 1 | 0 | 0 |
| 25-49% |  | 0 | 0 | 1 | 0 | 0 | 0 | 0 | 0 | 0 |
| 50%+ |  | 1 | 1 | 0 | 0 | 1 | 0 | 0 | 1 | 0 |
| Missing |  | 0 | 0 | 0 | 0 | 0 | 0 | 0 | 0 | 0 |
| **Pancreatic carcinoma** | 5 |  |  |  |  |  |  |  |  |  |
| <1% |  | 4 | 1 | 0 | 2 | 2 | 5 | 1 | 5 | 5 |
| 1-9% |  | 1 | 2 | 0 | 1 | 0 | 0 | 4 | 0 | 0 |
| 10-24% |  | 0 | 0 | 0 | 0 | 3 | 0 | 0 | 0 | 0 |
| 25-49% |  | 0 | 0 | 0 | 0 | 0 | 0 | 0 | 0 | 0 |
| 50%+ |  | 0 | 2 | 5 | 2 | 0 | 0 | 0 | 0 | 0 |
| Missing |  | 0 | 0 | 0 | 0 | 0 | 0 | 0 | 0 | 0 |
| **Cholangiocarcinoma** | 1 |  |  |  |  |  |  |  |  |  |
| <1% |  | 0 | 0 | 0 | 1 | 0 | 0 | 1 | 1 | 1 |
| 1-9% |  | 0 | 0 | 0 | 0 | 0 | 0 | 0 | 0 | 0 |
| 10-24% |  | 0 | 0 | 0 | 0 | 1 | 0 | 0 | 0 | 0 |
| 25-49% |  | 1 | 0 | 0 | 0 | 0 | 1 | 0 | 0 | 0 |
| 50%+ |  | 0 | 1 | 1 | 0 | 0 | 0 | 0 | 0 | 0 |
| Missing |  | 0 | 0 | 0 | 0 | 0 | 0 | 0 | 0 | 0 |
| **Hepatocellular carcinoma** | 4 |  |  |  |  |  |  |  |  |  |
| <1% |  | 4 | 4 | 4 | 4 | 4 | 4 | 4 | 4 | 4 |
| 1-9% |  | 0 | 0 | 0 | 0 | 0 | 0 | 0 | 0 | 0 |
| 10-24% |  | 0 | 0 | 0 | 0 | 0 | 0 | 0 | 0 | 0 |
| 25-49% |  | 0 | 0 | 0 | 0 | 0 | 0 | 0 | 0 | 0 |
| 50%+ |  | 0 | 0 | 0 | 0 | 0 | 0 | 0 | 0 | 0 |
| Missing |  | 0 | 0 | 0 | 0 | 0 | 0 | 0 | 0 | 0 |
| **Renal cell carcinoma ^b^** | 42 |  |  |  |  |  |  |  |  |  |
| <1% |  | 40 | 41 | 38 | 42 | 42 | 42 | 42 | 35 | 42 |
| 1-9% |  | 1 | 0 | 1 | 0 | 0 | 0 | 0 | 3 | 0 |
| 10-24% |  | 0 | 0 | 0 | 0 | 0 | 0 | 0 | 0 | 0 |
| 25-49% |  | 1 | 1 | 1 | 0 | 0 | 0 | 0 | 2 | 0 |
| 50%+ |  | 0 | 0 | 2 | 0 | 0 | 0 | 0 | 2 | 0 |
| Missing |  | 0 | 0 | 0 | 0 | 0 | 0 | 0 | 0 | 0 |
| **Breast carcinoma ^c^** | 27 |  |  |  |  |  |  |  |  |  |
| <1% |  | 27 | 25 | 6 | 27 | 27 | 26 | 20 | 25 | 27 |
| 1-9% |  | 0 | 0 | 0 | 0 | 0 | 0 | 2 | 1 | 0 |
| 10-24% |  | 0 | 0 | 0 | 0 | 0 | 0 | 0 | 1 | 0 |
| 25-49% |  | 0 | 0 | 0 | 0 | 0 | 0 | 1 | 0 | 0 |
| 50%+ |  | 0 | 0 | 21 | 0 | 0 | 1 | 4 | 0 | 0 |
| Missing |  | 0 | 2 | 0 | 0 | 0 | 0 | 0 | 0 | 0 |
| **Gynaecological (non-squamous) carcinomas ^d^** | 17 |  |  |  |  |  |  |  |  |  |
| <1% |  | 13 | 8 | 5 | 16 | 14 | 17 | 13 | 12 | 17 |
| 1-9% |  | 0 | 2 | 0 | 0 | 2 | 0 | 2 | 3 | 0 |
| 10-24% |  | 0 | 1 | 1 | 0 | 0 | 0 | 1 | 2 | 0 |
| 25-49% |  | 2 | 2 | 0 | 0 | 0 | 0 | 1 | 0 | 0 |
| 50%+ |  | 2 | 4 | 11 | 1 | 1 | 0 | 0 | 0 | 0 |
| Missing |  | 0 | 0 | 0 | 0 | 0 | 0 | 0 | 0 | 0 |
| **Prostatic carcinoma** | 11 |  |  |  |  |  |  |  |  |  |
| <1% |  | 10 | 5 | 11 | 10 | 10 | 11 | 11 | 9 | 11 |
| 1-9% |  | 1 | 2 | 0 | 1 | 0 | 0 | 0 | 2 | 0 |
| 10-24% |  | 0 | 0 | 0 | 0 | 1 | 0 | 0 | 0 | 0 |
| 25-49% |  | 0 | 1 | 0 | 0 | 0 | 0 | 0 | 0 | 0 |
| 50%+ |  | 0 | 3 | 0 | 0 | 0 | 0 | 0 | 0 | 0 |
| Missing |  | 0 | 0 | 0 | 0 | 0 | 0 | 0 | 0 | 0 |
| **Urothelial carcinoma** | 8 |  |  |  |  |  |  |  |  |  |
| <1% |  | 8 | 6 | 0 | 4 | 8 | 8 | 8 | 8 | 7 |
| 1-9% |  | 0 | 1 | 0 | 0 | 0 | 0 | 0 | 0 | 0 |
| 10-24% |  | 0 | 1 | 0 | 1 | 0 | 0 | 0 | 0 | 1 |
| 25-49% |  | 0 | 0 | 0 | 1 | 0 | 0 | 0 | 0 | 0 |
| 50%+ |  | 0 | 0 | 8 | 2 | 0 | 0 | 0 | 0 | 0 |
| Missing |  | 0 | 0 | 0 | 0 | 0 | 0 | 0 | 0 | 0 |
| **Squamous cell carcinoma ^e^** | 10 |  |  |  |  |  |  |  |  |  |
| <1% |  | 10 | 4 | 8 | 10 | 10 | 10 | 10 | 10 | 10 |
| 1-9% |  | 0 | 1 | 0 | 0 | 0 | 0 | 0 | 0 | 0 |
| 10-24% |  | 0 | 2 | 1 | 0 | 0 | 0 | 0 | 0 | 0 |
| 25-49% |  | 0 | 1 | 0 | 0 | 0 | 0 | 0 | 0 | 0 |
| 50%+ |  | 0 | 2 | 1 | 0 | 0 | 0 | 0 | 0 | 0 |
| Missing |  | 0 | 0 | 0 | 0 | 0 | 0 | 0 | 0 | 0 |
| **Adenoid cystic carcinoma ^f^** | 6 |  |  |  |  |  |  |  |  |  |
| <1% |  | 6 | 6 | 0 | 6 | 6 | 6 | 6 | 6 | 6 |
| 1-9% |  | 0 | 0 | 0 | 0 | 0 | 0 | 0 | 0 | 0 |
| 10-24% |  | 0 | 0 | 1 | 0 | 0 | 0 | 0 | 0 | 0 |
| 25-49% |  | 0 | 0 | 0 | 0 | 0 | 0 | 0 | 0 | 0 |
| 50%+ |  | 0 | 0 | 5 | 0 | 0 | 0 | 0 | 0 | 0 |
| Missing |  | 0 | 0 | 0 | 0 | 0 | 0 | 0 | 0 | 0 |
| **Thymoma ^g^** | 5 |  |  |  |  |  |  |  |  |  |
| <1% |  | 5 | 5 | 3 | 5 | 5 | 5 | 5 | 5 | 5 |
| 1-9% |  | 0 | 0 | 0 | 0 | 0 | 0 | 0 | 0 | 0 |
| 10-24% |  | 0 | 0 | 0 | 0 | 0 | 0 | 0 | 0 | 0 |
| 25-49% |  | 0 | 0 | 1 | 0 | 0 | 0 | 0 | 0 | 0 |
| 50%+ |  | 0 | 0 | 1 | 0 | 0 | 0 | 0 | 0 | 0 |
| Missing |  | 0 | 0 | 0 | 0 | 0 | 0 | 0 | 0 | 0 |
| **Thyroid carcinoma ^h^** | 3 |  |  |  |  |  |  |  |  |  |
| <1% |  | 3 | 3 | 1 | 3 | 3 | 3 | 3 | 2 | 1 |
| 1-9% |  | 0 | 0 | 0 | 0 | 0 | 0 | 0 | 0 | 0 |
| 10-24% |  | 0 | 0 | 0 | 0 | 0 | 0 | 0 | 0 | 0 |
| 25-49% |  | 0 | 0 | 0 | 0 | 0 | 0 | 0 | 0 | 0 |
| 50%+ |  | 0 | 0 | 2 | 0 | 0 | 0 | 0 | 1 | 2 |
| Missing |  | 0 | 0 | 0 | 0 | 0 | 0 | 0 | 0 | 0 |
| **Basal cell carcinoma** | 1 |  |  |  |  |  |  |  |  |  |
| <1% |  | 1 | 1 | 1 | 1 | 1 | 1 | 1 | 1 | 1 |
| 1-9% |  | 0 | 0 | 0 | 0 | 0 | 0 | 0 | 0 | 0 |
| 10-24% |  | 0 | 0 | 0 | 0 | 0 | 0 | 0 | 0 | 0 |
| 25-49% |  | 0 | 0 | 0 | 0 | 0 | 0 | 0 | 0 | 0 |
| 50%+ |  | 0 | 0 | 0 | 0 | 0 | 0 | 0 | 0 | 0 |
| Missing |  | 0 | 0 | 0 | 0 | 0 | 0 | 0 | 0 | 0 |

Abbreviations: CDH17, cadherin 17; CDX2, caudal type homeobox 2; CK, cytokeratin; GPA33, glycoprotein A33; MUC, mucin; SATB2, special AT-rich sequence-binding protein 2; TTF-1, thyroid transcription factor-1

^a^ all adenocarcinomas with gland formations and/or cribriform pattern with intestinal features

^b^ 33 clear cell, 4 papillary, and 5 other/indeterminate adenocarcinomas

^c^ 26 adenocarcinomas of ductal/no special type (whereof 1 mixed mucinous) and 1 malignant adenomyoepithelioma

^d^ 6 from uterus (5 endometroid adenocarcinomas, 1 carcinosarcoma), 8 from cervix (6 adenocarcinomas and 2 adenocarcinoma component of adenosquamous carcinomas), 2 from ovarium (1 clear cell and 1 mucinous adenocarcinoma), and 1 adenocarcinoma from vulva

^e^ 4 from tonsil, 3 from anus, 2 from esophagus, and 1 from uterine cervix

^f^ 4 from salivary glands and 2 from vulva

^g^ 1 type B1 and 4 type B3 (only epithelial cells evaluated)

^h^ 1 each of papillary, follicular, and anaplastic adenocarcinoma (the latter positive for SATB2 but negative for CK7 and TTF-1)

When not specified, cases were adenocarcinomas with morphological features typical of site of origin (e.g., gastrointestinal and prostatic carcinomas)
